# Teacher competency and work engagement among secondary school physical education teachers: the multiple mediating roles of occupational stress, emotional exhaustion, and professional achievement

**DOI:** 10.3389/fpsyt.2025.1530413

**Published:** 2025-02-03

**Authors:** Weisong Chen, Zhen Huang, Bo Peng, Lin Li, Jingsong Chen

**Affiliations:** ^1^ School of Sports Training, Chengdu Sport University, Chengdu, Sichuan, China; ^2^ School of Physical Education, Liaoning Normal University, Dalian, Liaoning, China; ^3^ Office of Scientific Research, Chengdu Vocational & Technical College, Chengdu, Sichuan, China

**Keywords:** teacher competency, work engagement, occupational stress, emotional exhaustion, professional achievement, secondary school teachers, physical education

## Abstract

**Objective:**

This study examined the relationships between teacher competency, occupational stress, emotional exhaustion, sense of professional achievement, and work engagement among secondary school physical education teachers. It also investigated demographic differences in these variables and tested the structural invariance of the proposed model across genders.

**Method:**

A total of 1,347 secondary school physical education teachers participated in the study, completing validated scales to measure the key constructs and providing demographic information such as gender, age, education level, professional title, and school location. Data were analyzed using descriptive statistics, t-tests, one-way ANOVA, correlation analysis, and structural equation modeling (SEM). Mediation effects were assessed through bootstrapping, and structural invariance was tested using multigroup SEM.

**Results:**

Demographic analyses revealed significant differences. Male teachers reported higher competency, professional achievement, and engagement, while female teachers experienced slightly greater emotional exhaustion. Younger teachers and those with lower professional titles reported higher occupational stress and emotional exhaustion. Rural and urban teachers exhibited comparable levels of most variables, except for urban teachers reporting slightly higher emotional exhaustion. Teacher competency directly influenced work engagement (28.27% of the total effect) and indirectly through occupational stress, emotional exhaustion, and professional achievement, with professional achievement being the strongest mediator (25.97% of the total effect). Sequential mediation pathways involving these factors were significant but weaker in magnitude. Structural invariance analysis confirmed that the model was consistent across genders, demonstrating robust applicability across male and female teacher populations.

**Conclusion:**

Teacher competency is a key driver of work engagement, operating through both direct and indirect mechanisms, particularly by alleviating occupational stress and enhancing professional achievement. These findings underscore the importance of interventions aimed at fostering teacher well-being, including stress management programs and professional development opportunities, while addressing demographic-specific needs. Future research should employ longitudinal designs and consider additional contextual factors to extend these findings.

## 1 Introduction

From a professional development perspective, the role of PE teachers extends beyond the classroom. These educators occupy a unique position where their influence directly shapes students’ physical and mental well-being, making them pivotal actors in addressing broader societal health challenges (1, 2). Secondary school students are particularly susceptible to forming health habits that persist into adulthood (3), highlighting the critical role of PE teachers in combating sedentary behaviors, obesity, and associated chronic conditions (4, 5). Consequently, teacher competency is not only a professional necessity but also a public health imperative.

However, the professional journey of PE teachers is fraught with challenges that can impede their ability to maintain engagement and deliver high-quality education. The multifaceted demands of teaching, coupled with the physical intensity of PE instruction, expose teachers to significant occupational stress (6–8). This stress can originate from excessive workloads, role ambiguity, limited institutional support, and the pressure to achieve both educational and public health outcomes (9, 10). Prolonged exposure to such stress often leads to emotional exhaustion, a hallmark of occupational burnout, which undermines professional efficacy and diminishes engagement with students (11, 12).

Conversely, professional achievement—a sense of accomplishment derived from fulfilling educational and developmental goals—has been identified as a critical protective factor in sustaining teacher engagement (13). Teachers who experience professional growth and recognition are more likely to exhibit resilience, enabling them to navigate occupational challenges effectively (9). For PE teachers, professional achievement also reinforces their role as advocates for health, bolstering their motivation to foster active, health-conscious behaviors in their students (14, 15). This dynamic interplay between stress, emotional exhaustion, and professional achievement represents a critical lens for understanding teacher engagement and well-being.

Despite significant advancements in understanding the factors influencing teachers’ professional well-being and performance (16), substantial gaps remain in the literature. Previous research has extensively documented the direct relationship between teacher competency and work engagement (17, 18), yet limited attention has been given to the mechanisms that mediate this relationship. Specifically, few studies have explored how occupational stress, emotional exhaustion, and professional achievement interact as mediators. Existing research often adopts a simplified approach, focusing on direct effects without fully examining the interconnections among these variables (19, 20). This oversimplification limits the understanding of the complex dynamics underlying teacher well-being and engagement, particularly in the high-demand context of PE teaching.

In addition, most studies on occupational stress and burnout have primarily focused on general classroom teachers (21, 22), often overlooking the unique pressures faced by PE teachers. Unlike their counterparts in other disciplines, PE teachers must navigate physically demanding work environments, manage large groups of active students, and align lessons with broader public health goals (23, 24). These distinct challenges exacerbate occupational stress, increase susceptibility to emotional exhaustion, and may hinder professional achievement. Yet, they remain underexplored in current literature, especially in the context of teacher competency and work engagement.

Another critical gap lies in understanding how teacher competency and its mediators vary across demographic factors such as gender, age, education level, professional title, and school location. For example, younger teachers may experience heightened stress due to less teaching experience, while urban school teachers might face greater emotional exhaustion due to higher administrative pressures. Similarly, differences in professional titles and education levels could influence teachers’ perceptions of their competency, stress levels, and professional achievement. However, few studies have systematically examined these demographic differences within the framework of teacher competency and work engagement. Addressing these demographic variations is essential for tailoring interventions to meet the specific needs of diverse teacher groups.

Furthermore, while professional achievement has been recognized as a critical resource that fosters motivation and engagement (13), its role as a buffering factor against occupational stress and emotional exhaustion remains underexplored. Few studies have examined how this sense of accomplishment interacts with negative psychological factors to sustain teacher engagement (25). The lack of a multidimensional approach that captures the simultaneous and interrelated effects of occupational stress, emotional exhaustion, and professional achievement further limits the practical implications of current research. Without such an integrative perspective, interventions often fail to address the complexity of these interactions, leading to suboptimal strategies for improving teacher well-being.

Addressing these gaps is crucial given the significant role of PE teachers in promoting adolescent health. Secondary school students are at a formative stage where lifelong habits related to physical activity, nutrition, and mental well-being are established (3). Engaged and resilient PE teachers play a pivotal role in fostering these habits, serving as role models who inspire healthy behaviors and teamwork. Conversely, teachers experiencing high levels of stress and exhaustion may struggle to motivate their students, potentially undermining educational and health outcomes (26).

This study aims to unpack the complex relationship between teacher competency and work engagement among secondary school PE teachers, focusing on the mediating roles of occupational stress, emotional exhaustion, and professional achievement. Furthermore, the study examines how these relationships differ across key demographic factors, including gender, age, education level, professional title, and school location. By situating this analysis within the broader framework of teacher professional development, the research highlights the dual significance of supporting teachers’ occupational health: not only as a foundation for their career sustainability but also as a prerequisite for promoting adolescent health. The findings are expected to provide actionable insights for education policymakers, school administrators, and teacher training programs, emphasizing the importance of fostering teacher well-being as a cornerstone for societal health and educational advancement.

## 2 Literature review and theoretical framework

### 2.1 Teacher competency

Teacher competency forms the foundation of effective teaching and professional development, especially in the demanding field of secondary school physical education (PE). It involves the integration of knowledge, skills, attitudes, and behaviors necessary to achieve educational objectives (27). Beyond instructional proficiency, competency encompasses the ability to inspire students and foster their physical and emotional growth (28–30). For PE teachers, this includes a deep understanding of sports science, physical health, and pedagogy, as well as interpersonal skills and emotional intelligence to build positive relationships with students.

The significance of teacher competency extends beyond student outcomes, influencing teachers’ professional well-being and resilience (31). Competent teachers are more confident and effective in managing challenges, which enhances their job satisfaction and buffers against occupational stress (32). In secondary school PE settings, where physical demands, large class sizes, and varying student attitudes toward exercise are common, teacher competency plays a critical role in maintaining classroom dynamics and promoting student engagement. For instance, PE teachers who design inclusive, engaging activities tailored to diverse needs are better equipped to address behavioral issues and foster a supportive learning environment (33).

Additionally, teacher competency is essential for long-term professional sustainability. Competent educators are more likely to pursue continuous learning opportunities, such as advanced certifications or professional development workshops (34, 35). These activities not only improve teaching effectiveness but also reinforce professional identity, contributing to career satisfaction and reducing burnout.

Furthermore, teacher competency holds a pivotal role in promoting adolescent health. Adolescents are at a formative stage where lifelong habits related to physical activity, nutrition, and mental well-being are established (36). Competent PE teachers serve as role models, encouraging students to adopt healthier lifestyles (37, 38). Through engaging and well-structured lessons, they can dispel fitness misconceptions, address barriers such as low self-confidence, and promote teamwork and stress management skills (39).

Despite its importance, teacher competency often faces systemic challenges. PE is frequently undervalued compared to core academic subjects, resulting in limited institutional support and resources (40). This undervaluation, combined with the profession’s physical and emotional demands, hinders the development and application of teacher competencies (41, 42). Addressing these issues requires coordinated efforts from policymakers and educational leaders to prioritize professional development, allocate resources, and recognize the critical role of PE in holistic education.

Teacher competency is, therefore, a cornerstone of both individual and societal well-being. For teachers, it enhances their ability to navigate professional complexities and achieve fulfillment. For students, it ensures access to quality education that nurtures physical and mental health, preparing them for lifelong well-being. Strengthening teacher competency through targeted interventions and systemic support can create a lasting positive impact on both teachers and the communities they serve.

### 2.2 Work engagement

Work engagement, a positive and fulfilling psychological state characterized by vigor, dedication, and absorption, is increasingly recognized as a critical determinant of teachers’ professional well-being and performance (43, 44). For secondary school physical education (PE) teachers, work engagement is not just a personal trait but a key driver of their ability to motivate students, deliver effective lessons, and foster an environment conducive to learning and physical development (45, 46). Unlike job satisfaction, which reflects general contentment with work, work engagement emphasizes active involvement and enthusiasm in fulfilling professional responsibilities.

The concept of work engagement is grounded in the Job Demands-Resources (JD-R) Model, which posits that engagement arises from the dynamic balance between job demands and resources (47). In the context of PE teachers, job demands include physical exertion, emotional labor in managing student relationships, and administrative responsibilities (48). Excessive demands can lead to occupational stress and emotional exhaustion, diminishing teachers’ capacity to remain engaged (33). Conversely, access to resources—such as supportive leadership, professional development opportunities, and a collaborative school culture—can buffer against these challenges, fostering resilience and sustaining engagement (49, 50).

Engaged teachers demonstrate high levels of energy and motivation, even when confronted with challenges. For PE teachers, this translates to maintaining their enthusiasm for physical activity and inspiring the same enthusiasm in students (51). Work engagement enables teachers to create inclusive and dynamic learning environments that encourage student participation, nurture a love for physical fitness, and promote teamwork and cooperation (5). These outcomes are particularly impactful for adolescents, who often face barriers to physical activity, such as low self-efficacy, social pressures, or limited access to recreational opportunities.

The benefits of work engagement extend beyond immediate classroom outcomes to encompass broader professional and societal impacts. Engaged teachers are more likely to experience job satisfaction, professional growth, and reduced burnout, contributing to career sustainability (52, 53). Moreover, their positive energy and dedication often influence colleagues, fostering a cohesive and supportive school environment (54). For PE teachers, whose work aligns with public health objectives, high levels of engagement can also address societal challenges such as adolescent physical inactivity and obesity (5).

However, sustaining work engagement among PE teachers is not without challenges. The physically demanding nature of their role, coupled with limited recognition of PE’s importance in academic curricula and potential resource constraints, often hinders their ability to maintain engagement (40). Emotional exhaustion, a key component of burnout, is particularly detrimental, as it depletes the psychological resources necessary for sustained engagement (55, 56). This underscores the critical role of fostering professional achievement—a sense of accomplishment and recognition in one’s role—as a buffer against exhaustion and a driver of motivation.

Work engagement is thus a dynamic construct reflecting the interplay between individual, organizational, and societal factors. For secondary school PE teachers, it represents not only a cornerstone of effective teaching and professional well-being but also a vital contributor to public health advocacy. Enhancing work engagement requires interventions that address occupational stress, strengthen support systems, and recognize the unique contributions of PE teachers to student and community health (57). In doing so, educational institutions can ensure not only the sustainability of teachers’ professional engagement but also a lasting positive impact on student outcomes and societal well-being.

### 2.3 Occupational stress, emotional exhaustion, and professional achievement

Occupational stress, emotional exhaustion, and professional achievement are interrelated constructs that profoundly influence teachers’ professional well-being and work engagement. Each variable represents a distinct but interconnected mechanism that mediates how professional competencies impact teaching performance and personal fulfillment.

Occupational stress is defined as the psychological response to work demands that exceed an individual’s resources or coping capacity (49, 58). It is a pervasive challenge in the teaching profession, particularly for physical education (PE) teachers, who often navigate physically intensive workloads, large and active student groups, and the broader responsibility of promoting public health goals through physical activity. Prolonged exposure to occupational stress can deplete teachers’ psychological and physical resources, leading to reduced focus, energy, and commitment in their roles (59, 60).

Emotional exhaustion, a core component of burnout, is one of the most immediate consequences of prolonged occupational stress. It refers to a state of chronic emotional and physical depletion that diminishes teachers’ ability to engage with their work and students effectively (55, 61). PE teachers, given the dual physical and emotional demands of their role, are particularly vulnerable to emotional exhaustion (62). When emotionally depleted, teachers may struggle to maintain the enthusiasm and energy required to inspire students, ultimately compromising both teaching effectiveness and personal well-being (56).

In contrast, professional achievement serves as a critical resource that buffers against stress and exhaustion, enhancing teachers’ resilience and motivation (63, 64). Professional achievement encompasses the sense of fulfillment derived from successfully meeting professional goals, gaining recognition, and witnessing the tangible outcomes of one’s efforts (65). For PE teachers, these achievements may manifest as improved student fitness, positive attitudes toward health, or overcoming behavioral challenges. The sense of accomplishment associated with professional achievement reinforces intrinsic motivation, empowering teachers to persist in the face of professional challenges (66–68).

The dynamic relationships among these variables are particularly important in understanding their combined effects on teacher engagement and well-being. Occupational stress often acts as a precursor to emotional exhaustion, draining the psychological and physical resources needed to sustain engagement (13). Emotional exhaustion, in turn, undermines professional achievement by reducing teachers’ capacity to perform effectively and recognize the value of their contributions (69). Conversely, strong professional achievement can mitigate the effects of both stress and exhaustion, serving as a motivational resource that fosters resilience and sustains engagement (70, 71).

These constructs also play a pivotal role in broader educational outcomes. High levels of occupational stress and emotional exhaustion are associated with diminished teaching quality and reduced capacity to foster students’ physical and mental well-being. Conversely, professional achievement not only enhances teachers’ psychological health but also amplifies their positive influence on students (72, 73). This highlights the importance of understanding and addressing these interconnected variables to support teacher well-being and optimize educational outcomes.

The intricate relationships among occupational stress, emotional exhaustion, and professional achievement emphasize the critical role these variables play in mediating the effects of teacher competency on work engagement. To further explore these dynamics, this study proposes a series of hypotheses aimed at identifying the direct and indirect pathways through which teacher competency influences work engagement.

**Direct Effect:**

H1: Teacher competency has a positive direct effect on work engagement, representing the strongest and most immediate pathway.

**Simple Mediation Pathways:**

H2: Occupational stress mediates the relationship between teacher competency and work engagement, highlighting the role of stress management in enhancing engagement.

H3: Emotional exhaustion mediates the relationship between teacher competency and work engagement, reflecting the impact of emotional resilience on professional commitment.

H4: Professional achievement mediates the relationship between teacher competency and work engagement, emphasizing the motivational role of accomplishment in sustaining engagement.

**Chain Mediation Pathways:**

H5: Teacher competency positively influences work engagement through the sequential mediation of occupational stress and emotional exhaustion.

H6: Teacher competency positively influences work engagement through the sequential mediation of occupational stress and professional achievement.

H7: Teacher competency positively influences work engagement through the sequential mediation of emotional exhaustion and professional achievement.

H8: Teacher competency positively influences work engagement through the combined chain mediation of occupational stress, emotional exhaustion, and professional achievement.

These hypotheses offer a structured approach to examining the multifaceted effects of teacher competency on work engagement, encompassing both direct and indirect mechanisms. This framework not only reflects the complex nature of the relationships among occupational stress, emotional exhaustion, and professional achievement but also provides a basis for the empirical testing and theoretical analysis to follow. The next section will build on these insights with specific theoretical perspectives for further analysis.

### 2.4 Theoretical framework

This study adopts the Job Demands-Resources (JD-R) Model as the primary theoretical framework to examine the relationships among teacher competency, occupational stress, emotional exhaustion, professional achievement, and work engagement. The JD-R model posits that work engagement arises from the balance between job demands and job resources (47). Job demands refer to the physical, emotional, and cognitive efforts required to fulfill work responsibilities, which, when excessive, lead to stress and resource depletion. Conversely, job resources represent factors that enhance personal development, mitigate the effects of demands, and foster motivation and engagement (59, 74). These resources include professional achievement, supportive leadership, and access to development opportunities (10).

For secondary school PE teachers, job demands often include the physically intensive nature of their work, the need to manage large and active groups of students, and the responsibility to meet broader public health goals through physical education (75). These demands can deplete their psychological and physical energy, contributing to occupational stress and emotional exhaustion. The JD-R model highlights that excessive demands trigger the health impairment process, where sustained exposure to stressors drains teachers’ energy and reduces their capacity to engage effectively in their roles (47). Emotional exhaustion, a key dimension of burnout, represents the depletion of emotional resources caused by prolonged exposure to occupational stress and is particularly relevant to PE teachers due to the dual physical and emotional demands of their roles (76, 77).

At the same time, job resources play a pivotal role in counterbalancing these demands and supporting work engagement. Professional achievement, defined as the sense of accomplishment derived from meeting professional goals and witnessing positive outcomes, is one of the most critical resources in this context (13). It serves as a motivational factor that fosters resilience, reinforces intrinsic motivation, and enhances teachers’ ability to remain engaged despite challenges. For PE teachers, professional achievement can manifest in improved student fitness, positive changes in student attitudes toward physical health, and the resolution of behavioral challenges (78, 79). These experiences not only provide a sense of purpose but also buffer against the negative effects of occupational stress and emotional exhaustion.

The JD-R model further emphasizes the motivational process (47), where job resources directly enhance work engagement by fostering vigor, dedication, and absorption. In the context of PE teaching, engagement is critical for sustaining enthusiasm for physical activity and inspiring the same in students. Teachers with higher levels of engagement are better positioned to create inclusive, dynamic learning environments that encourage student participation, foster teamwork, and promote lifelong healthy behaviors (80). This motivational process underscores the importance of strengthening job resources, such as professional achievement, to sustain engagement and support teacher well-being.

In addition to the JD-R model, this study draws on Burnout Theory to complement its understanding of emotional exhaustion. Burnout Theory identifies emotional exhaustion as a central component of burnout and highlights its role in diminishing work engagement and professional effectiveness (81). Teachers experiencing emotional exhaustion often struggle to maintain the energy and enthusiasm required to inspire students and fulfill their professional responsibilities. However, Burnout Theory also underscores the importance of fostering personal accomplishment, which aligns closely with the concept of professional achievement, as a critical resource that mitigates the negative effects of emotional exhaustion (82). By integrating insights from Burnout Theory, this study provides a more nuanced understanding of the mechanisms through which occupational stress and emotional exhaustion impact work engagement, while reinforcing the role of professional achievement as a protective factor.

**Figure 1:**
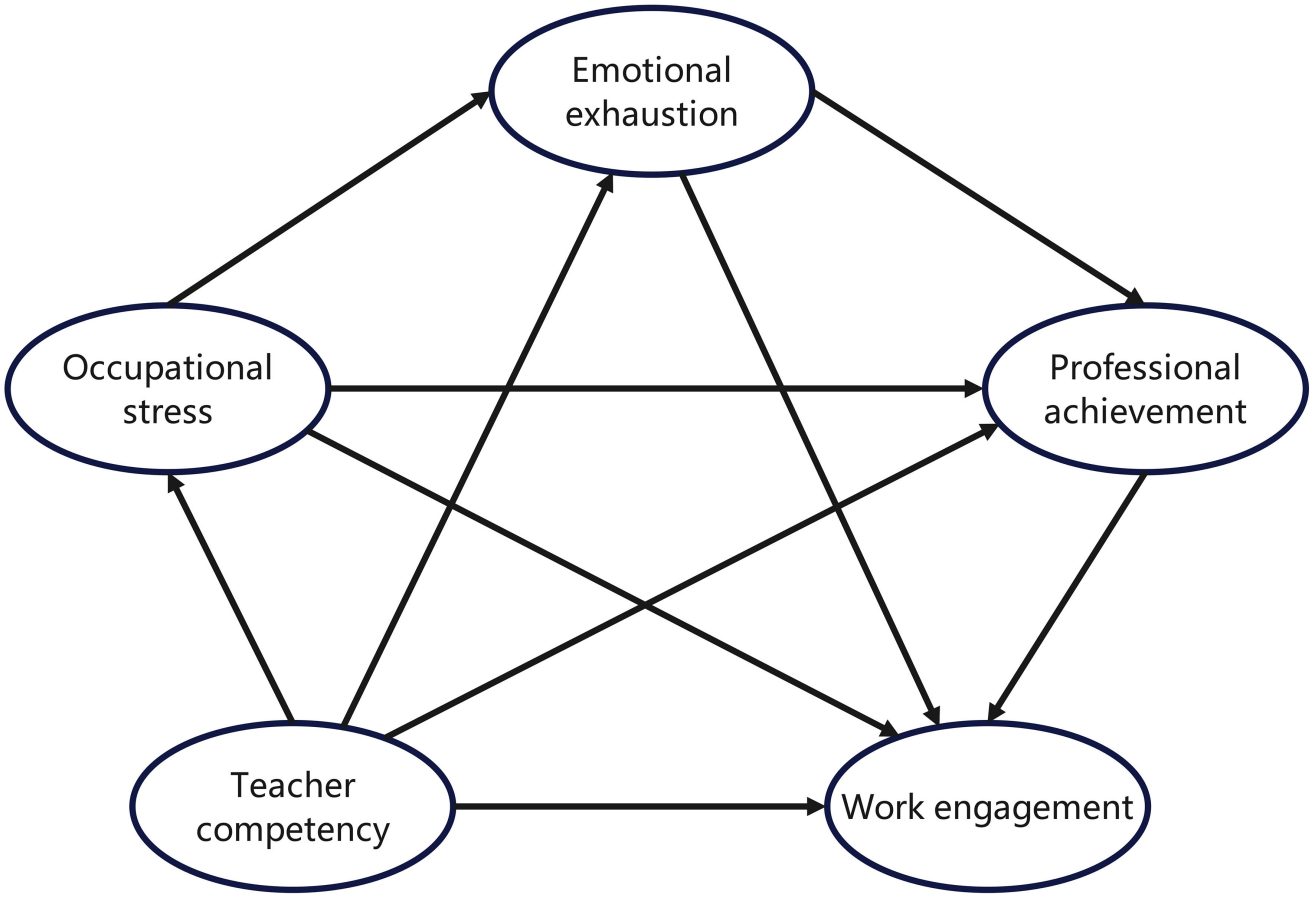
Conceptual Framework.

Together, the JD-R model and Burnout Theory form a comprehensive framework for exploring how teacher competency influences work engagement through occupational stress, emotional exhaustion, and professional achievement. Based on these theoretical foundations, this study develops an integrated conceptual model (**Figure 1**) that visually illustrates the direct effects, simple mediation pathways, and sequential chain mediation pathways among key constructs. This model serves as the basis for empirical testing and highlights the dynamic interplay of job demands, resources, and outcomes in secondary school PE teaching.

## 3 Materials and methodology

### 3.1 Participants and data

#### 3.1.1 Sample size justification

To ensure the robustness and reliability of this study, a sample size estimation was conducted using GPower software and established guidelines commonly applied in social science research. A power analysis was performed in G*Power 3.1 for multiple regression analysis, involving the following variables: Teacher Competency (independent variable), Work Engagement (dependent variable), Occupational Stress (mediator), Emotional Exhaustion (mediator), and Professional Achievement (mediator). Assuming a medium effect size (*f* ² = 0.15), a significance level of α = 0.05, and a desired statistical power of 1-β = 0.80, the analysis indicated that a minimum of 389 participants would be required to detect statistically significant effects for each hypothesized model.

Additionally, the participant-to-item ratio guideline for survey-based studies in social sciences was referenced, which recommends 5 to 10 participants per survey item. The survey instrument used in this study comprises 116 items, including the Teacher Competency scale (22 items), the Work Engagement scale (17 items), the Occupational Stress scale (46 items), the Emotional Exhaustion scale (6 items), the Professional Achievement scale (20 items), and 5 demographic items (gender, age, teaching experience, school location, and education level). Based on this guideline, a sample size of approximately 580 to 1,160 participants is recommended.

To ensure sufficient statistical power and generalizability, 1,500 questionnaires were distributed to secondary school physical education teachers. After data cleaning to remove incomplete or invalid responses, 1,347 valid questionnaires were retained, achieving an effective response rate of 89.8%. This sample size exceeds both the requirements of the G*Power analysis and the participant-to-item ratio guideline, ensuring robust statistical power, generalizability, and reliability across diverse demographic subgroups.

#### 3.1.2 Participant selection process

Participants were selected using a stratified random sampling technique to ensure representation across different regions and demographic groups, thereby minimizing selection bias. The sampling frame consisted of frontline physical education teachers from 15 provinces and cities across China.

The randomization process was conducted in multiple stages. First, stratification was applied at the regional level, with schools randomly selected within each stratum. Subsequently, frontline physical education teachers were randomly chosen from the selected schools to participate. This multi-level randomization ensured that the sample represented frontline physical education teachers from various regions of China. The selection process included the following specific steps:

##### 3.1.2.1 Regional stratification

Based on China’s socioeconomic and cultural diversity, the country was divided into five geographic strata. Three provinces or cities were selected from each region to ensure balanced representation. **Table 1** provides details on the regions and the selected provinces.

**Table 1:** Stratification by region and province in the sampling frame.

Region	Province
Eastern China	Shanghai, Jiangsu, Shandong
Central China	Henan, Hubei, Hunan
Western China	Sichuan, Guizhou, Chongqing
Southern China	Guangdong, Guangxi, Hainan
Northern China	Beijing, Shaanxi, Liaoning

##### 3.1.2.2 Random selection of schools

Within each geographic stratum, schools were selected using a random number generator. The number of schools chosen from each region was proportionate to the population of secondary school physical education teachers in that area, ensuring balanced representation. For example, if a region contained 20% of the total population of secondary school PE teachers, 20% of the schools in that region were selected.

##### 3.1.2.3 Random selection of schools

In each selected school, physical education teachers were randomly chosen from a list of frontline PE teachers in grades seven through nine, using a random number generator. This process ensured that each PE teacher had an equal chance of being selected, further reducing selection bias.

#### 3.1.3 Data collection methods

##### 3.1.3.1 Training of data collectors

Prior to the distribution of questionnaires, all research team members received comprehensive training. This training covered the study’s objectives, the importance of random sampling, and standardized instructions for distributing the questionnaires to ensure consistency in the process.

##### 3.1.3.2 Questionnaire distribution

Questionnaires were distributed on regular school days, supervised by research team members to ensure that physical education teachers completed the questionnaires independently and without external interference. Completed questionnaires were collected immediately to ensure prompt and accurate data recovery.

##### 3.1.3.3 Confidentiality and informed consent

This study strictly adhered to the Helsinki Declaration and relevant national and institutional guidelines. Ethical approval was obtained from the Ethics Committee of Chengdu Sport University (Approval No. CTYLL2024013). As all participants were adults, verbal consent was obtained from each participant before participation, ensuring compliance with ethical standards. Data analysis was conducted anonymously to protect participant privacy and maintain data confidentiality.

#### 3.1.4 Data processing

The survey was conducted from March 1, 2024, to July 1, 2024, with a total of 1,500 questionnaires distributed. After excluding invalid questionnaires due to incorrect entries, omissions, and fixed responses, 1,347 valid questionnaires were collected, resulting in an effective response rate of 89.80%. Invalid questionnaires were screened out based on predefined criteria (including incomplete responses and inconsistent answers) to ensure data quality and completeness. Basic information of the respondents is shown in **Table 2**.

**Table 2:** The sample information.

Basic information	Category	Frequency	Percentage
Gender	Male	659	48.92
	Female	688	51.08
Age	≤30	339	25.17
	31-40	552	40.98
	41-50	398	29.55
	>50	58	4.31
Education level	Junior college and below	323	23.98
	Bachelor	695	51.60
	Graduate	329	24.42
Professional title	No title	622	46.18
	Primary	497	36.90
	Intermediate	190	14.11
	Senior	38	2.82
School location	Rural	679	50.41
	Urban	668	49.59

### 3.2 Measurement tools

Teacher Competency was assessed using a scale with 22 items across five dimensions: research and innovation ability (5 items), information-gathering and learning ability (3 items), teaching and organizational ability (5 items), professional knowledge (3 items), and personal characteristics (6 items) (83). Participants responded on a 5-point Likert scale, ranging from 1 (very poor) to 5 (very good). This scale comprehensively captures the multidimensional nature of teacher competency and has demonstrated high reliability and validity in previous research.

Work Engagement was measured using a scale with 17 items divided into three dimensions: vigor (6 items), dedication (5 items), and absorption (6 items) (84). Responses were recorded on a 5-point Likert scale, ranging from 1 (almost never) to 5 (always). This scale is a validated and widely applied instrument for assessing teachers’ engagement in their professional roles.

Occupational Stress was evaluated using a 46-item scale across six dimensions: examination pressure (9 items), student factors (13 items), need for self-development (9 items), family and interpersonal relationships (6 items), workload (5 items), and career expectations (4 items) (85). The items were rated on a 5-point Likert scale, from 1 (no stress) to 5 (high stress). This instrument provides a detailed assessment of the sources of occupational stress and has shown strong reliability in educational contexts.

Emotional Exhaustion was assessed using a scale with 6 items (86). Emotional exhaustion is defined as a psychological manifestation of stress and a core dimension of job burnout, often occurring alongside physical fatigue. It typically arises when emotional and psychological resources are depleted, leading to potential increases in turnover intentions and decreases in job performance levels. Participants rated their agreement with statements on a 5-point Likert scale ranging from 1 (strongly disagree) to 5 (strongly agree). This scale has demonstrated high reliability and validity in prior research, making it a robust measure of emotional exhaustion in educational contexts.

Sense of Professional Achievement was measured using a 20-item scale divided into two dimensions: experience of success (8 items) and self-efficacy (12 items) (87). Responses were recorded on a 5-point Likert scale, from 1 (strongly disagree) to 5 (strongly agree). This instrument assesses the intrinsic satisfaction and accomplishments perceived by teachers in their professional roles and has demonstrated high reliability and validity in prior studies.

Reverse-scored items across all scales were adjusted to ensure consistency in positive scoring. The use of well-validated instruments for all variables ensures the reliability and validity of the measurements in this study. **Table 3** summarizes the scale indicators used in the study.

**Table 3:** Scales used in this study.

Scale	Author (Year)	Item quantity	Scoring	Dimensions
Teacher competency	Changcheng Zhang (2011) (83)	22	5	Research and innovation ability, information-gathering and learning ability, teaching and organizational ability, professional knowledge, personal characteristics
Occupational stress	Congshu Zhu et al. (2002) (85)	46	5	Examination pressure, student factors, need for self-development, family and interpersonal relationships, workload, career expectations
Emotional exhaustion	Samuel Aryee et al. (2008) (86)	6	5	–
Sense of professional achievement	Rui Deng (2013) (87)	20	5	Experience of success, self-efficacy
Work engagement	Schaufeli W. B. et al. (2022) (84)	17	5	Vigor, dedication, absorption

### 3.3 Data analysis procedure

To assess potential common method bias, Harman’s single-factor test was conducted using principal component analysis (PCA) (88). All survey items were loaded into an unrotated factor solution, and the proportion of variance explained by the largest factor was evaluated against the critical threshold of 40%. This ensured that common method bias was minimized and did not interfere with subsequent analyses.

Descriptive statistics, including means and standard deviations for all key variables, were calculated to provide an overview of the sample’s characteristics and the data distribution. These metrics offered foundational insights for further statistical testing and ensured the data met the basic requirements for subsequent analyses. The reliability of each construct was evaluated using Cronbach’s alpha coefficients, with thresholds set at 0.70 for acceptable reliability and 0.80 for strong internal consistency (89).

Confirmatory Factor Analysis (CFA) was conducted to validate the measurement model. Individual CFAs were performed to assess the unidimensionality of each construct, ensuring that all factor loadings exceeded 0.50. A multi-factor CFA was then conducted to evaluate the discriminant and convergent validity of the constructs. Model fit was assessed using the following indices and corresponding thresholds: χ²/*df* (Chi-Square/df): < 3.00 (acceptable fit), CFI (Comparative Fit Index): ≥ 0.90 (good fit), TLI (Tucker-Lewis Index): ≥ 0.90 (good fit), RMSEA (Root Mean Square Error of Approximation): ≤ 0.08 (acceptable fit), SRMR (Standardized Root Mean Square Residual): ≤ 0.08 (acceptable fit) (90).

Correlation analysis was conducted to examine the relationships among key variables. Pearson correlation coefficients were used to determine the strength and direction of these associations, with coefficients interpreted as follows: 0.10–0.29 (weak), 0.30–0.49 (moderate), and ≥ 0.50 (strong). This analysis provided preliminary evidence for the structural relationships to be tested in subsequent modeling (89).

To explore differences in key variables across demographic subgroups, independent samples t-tests and one-way ANOVA were conducted. For significant ANOVA results, *post-hoc* tests such as Tukey’s HSD were performed to identify specific group differences. These tests were used to examine demographic factors such as gender, age, education level, professional title, and school location, helping to understand the influence of these factors on key constructs (91).

Structural Equation Modeling (SEM) was employed to evaluate the hypothesized direct and mediation effects. Model fit was assessed using the following indices and thresholds: χ²/df: < 3.00 (acceptable fit), CFI: ≥ 0.95 (excellent fit), TLI: ≥ 0.95 (excellent fit), RMSEA: ≤ 0.06 (excellent fit), SRMR: ≤ 0.08 (acceptable fit). Path analysis within the SEM framework examined both direct effects (e.g., Teacher Competency → Work Engagement) and mediation effects through constructs such as Occupational Stress, Emotional Exhaustion, and Sense of Professional Achievement. Standardized coefficients (β) were reported to quantify the strength of these relationships, with significance determined at p < 0.05 (92).

To confirm mediation effects, bootstrapping with 2,000 resamples was conducted. Bias-corrected confidence intervals (95%) were generated, and mediation effects were considered significant if the confidence intervals did not include zero. Standardized coefficients were used to calculate effect sizes for direct, indirect, and total effects, providing a comprehensive understanding of the pathways in the model (93).

Finally, multigroup structural equation modeling (MGSEM) was performed to test structural invariance across gender groups. Measurement invariance was assessed by comparing unconstrained and constrained models, with changes in CFI (ΔCFI ≤ 0.01) used as the primary criterion for invariance. This analysis ensured that the structural relationships in the model were consistent across demographic groups (94).

## 4 Result

### 4.1 Common method bias and multicollinearity test

To assess the potential impact of common method bias, a principal component analysis was conducted using Harman’s single-factor test. Sixteen factors with eigenvalues greater than 1 were extracted, with the largest factor accounting for 25.25% of the total variance, which is below the critical threshold of 40%. This indicates that common method bias is not a significant concern in this study, suggesting minimal interference with subsequent analyses.

### 4.2 Descriptive statistics, reliability, and construct validity of the measurement model

The descriptive statistics, internal consistency reliability, and fit indices for the measurement model are presented in **Table 4**. The mean scores (M) for the key constructs ranged from 2.788 (Emotional Exhaustion) to 3.565 (Work Engagement), indicating moderate to high levels across the sample. The standard deviations (SD) ranged from 0.612 to 0.797, suggesting acceptable variability among responses.

**Table 4:** Descriptive statistics, internal consistency reliability, and fit indices for confirmatory factor analysis (CFA) of key variables.

Variable	M	SD	α	CFI	TLI	SRMR	RMSEA (90%CI)
Teacher competency	3.406	0.625	0.925	0.959	0.953	0.027	0.045 (0.042-0.048)
Occupational stress	3.039	0.612	0.961	0.981	0.980	0.021	0.021 (0.020-0.023)
Emotional exhaustion	2.788	0.797	0.875	0.999	0.998	0.001	0.018 (0.000-0.038)
Sense of professional achievement	3.207	0.640	0.933	0.977	0.974	0.022	0.038 (0.035-0.042)
Work engagement	3.565	0.626	0.915	0.968	0.963	0.025	0.046 (0.042-0.051)

#### 4.2.1 Reliability

The Cronbach’s alpha (α) values for all constructs exceeded 0.87, with the highest reliability observed for Occupational Stress (α = 0.961) and the lowest for Emotional Exhaustion (α = 0.875). These results indicate excellent internal consistency across all scales, meeting the standard reliability threshold (α > 0.70).

#### 4.2.2 Construct validity

Confirmatory factor analysis (CFA) results demonstrated satisfactory model fit for all constructs, with fit indices meeting recommended thresholds. Specifically, Comparative Fit Index (CFI) values ranged from 0.959 to 0.999, Tucker-Lewis Index (TLI) values ranged from 0.953 to 0.998, and Standardized Root Mean Square Residual (SRMR) values ranged from 0.001 to 0.027. The Root Mean Square Error of Approximation (RMSEA) values ranged from 0.018 (Emotional Exhaustion) to 0.046 (Work Engagement), all within the acceptable range (< 0.08). These results indicate strong construct validity for the measurement model.

#### 4.2.3 Model comparison

**Table 5** compares the goodness-of-fit indices for the five-factor model and alternative models. The five-factor model (Teacher Competency, Occupational Stress, Emotional Exhaustion, Sense of Professional Achievement, and Work Engagement) exhibited the best fit to the data, with χ² = 441.461, df = 110, CFI = 0.966, TLI = 0.958, SRMR = 0.041, and RMSEA = 0.047 (90% CI: 0.043–0.052). In contrast, alternative models (four-factor, three-factor, two-factor, and one-factor) demonstrated significantly poorer fit, with higher χ² values and lower CFI and TLI values. The Δχ² test confirmed that the five-factor model significantly outperformed the alternative models (p < 0.05 for all comparisons).

**Table 5:** Comparative fit indices for alternative factor structures of key constructs (teacher competency, occupational stress, emotional exhaustion, sense of professional achievement, and work engagement).

Model	Factor	χ 2	*df*	△χ2 (△*df*)	CFI	TLI	SRMR	RMSEA (90%CI)
Five-factor model	TEC, OCS, EME, SPA, WOE	441.461	110	–	0.966	0.958	0.041	0.047 (0.043-0.052)
Four-factor model	TEC+ OCS, EME, SPA, WOE	2112.615	114	1671.154 (4)	0.793	0.753	0.095	0.114 (0.110-0.118)
Three-factor model	TEC+ OCS+ EME, SPA, WOE	2173.577	116	1732.116 (6)	0.787	0.750	0.096	0.115 (0.111-0.119)
Two-factor model	TEC+ OCS+ EME+ SPA, WOE	2301.252	118	1859.791 (8)	0.774	0.739	0.095	0.117 (0.113-0.121)
One-factor model	TEC+ OCS+ EME+ SPA+ WOE	2621.754	119	2180.293 (9)	0.741	0.704	0.098	0.125 (0.121-0.129)

TEC, Teacher competency; OCS, Occupational stress; EME, Emotional exhaustion; SPA, Sense of professional achievement; WOE, Work engagement. All △χ2 passed the significance test at 0.05 level.

These findings provide robust evidence for the reliability, discriminant validity, and overall construct validity of the measurement model, supporting the appropriateness of the five-factor structure for subsequent analysis.

### 4.3 Correlation analysis among key variables

**Figure 2:**
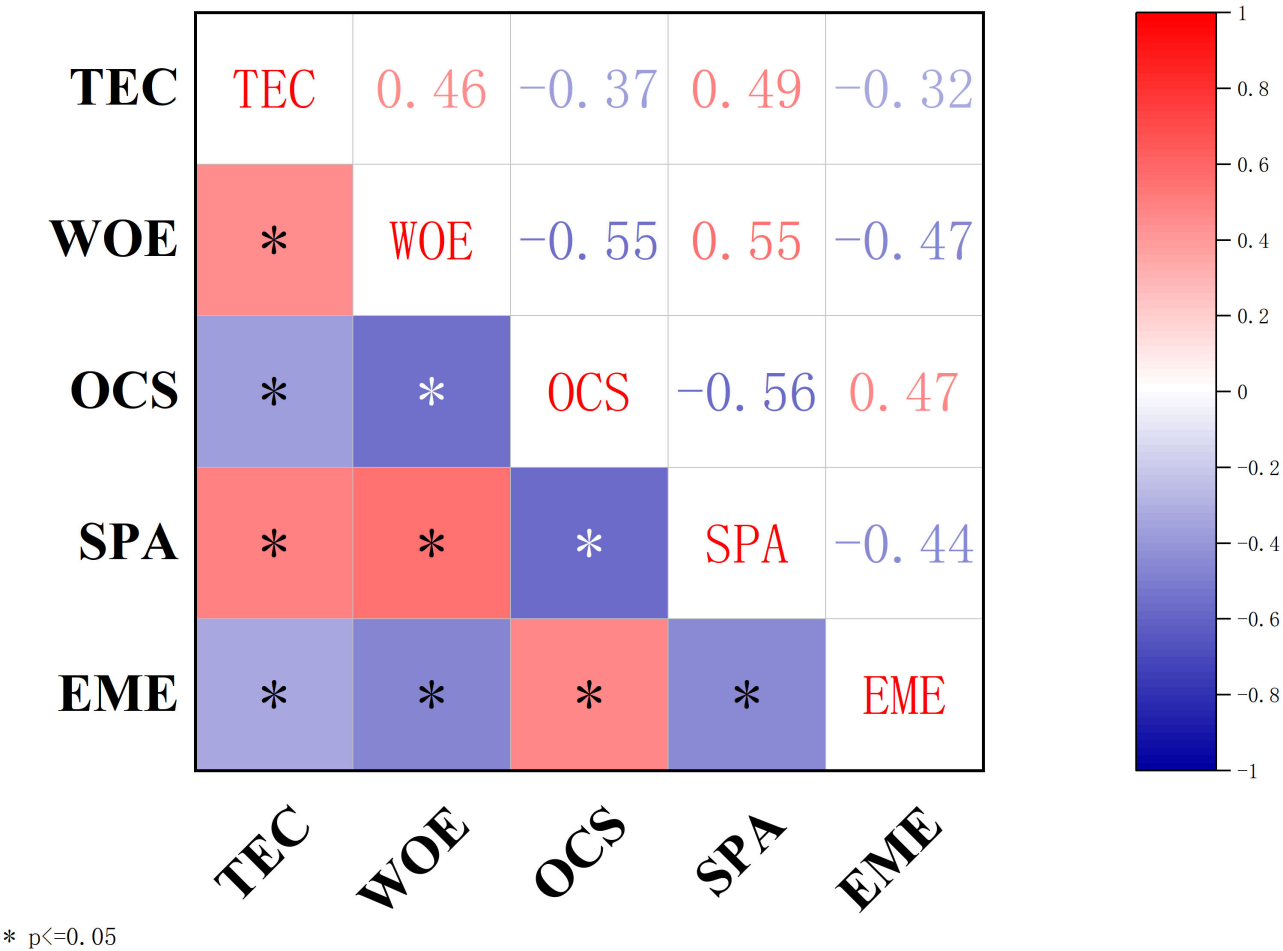
Correlation matrix of key variables. TEC, Teacher competency; OCS, Occupational stress; EME, Emotional exhaustion; SPA, Sense of professional achievement; WOE, Work engagement.

The correlation matrix of key variables, as shown in **Figure 2**, highlights significant relationships among Teacher Competency (TEC), Occupational Stress (OCS), Emotional Exhaustion (EME), Sense of Professional Achievement (SPA), and Work Engagement (WOE). These findings offer preliminary evidence for the hypothesized relationships in the structural model.

Teacher Competency (TEC) displayed a significant positive correlation with Work Engagement (WOE; r = 0.46) and Sense of Professional Achievement (SPA; r = 0.49), indicating that higher competency is associated with increased professional achievement and engagement. Conversely, TEC was negatively correlated with Occupational Stress (OCS; r = -0.37) and Emotional Exhaustion (EME; r = -0.32), suggesting its protective role in reducing stress and exhaustion.

Occupational Stress (OCS) was negatively correlated with both Work Engagement (WOE; r = -0.55) and Sense of Professional Achievement (SPA; r = -0.56). In addition, OCS showed a positive correlation with Emotional Exhaustion (EME; r = 0.47), underscoring its detrimental effects on teachers’ psychological well-being and professional outcomes.

Emotional Exhaustion (EME) demonstrated a negative correlation with both Work Engagement (WOE; r = -0.47) and Sense of Professional Achievement (SPA; r = -0.44), further highlighting its adverse impact on motivation and professional satisfaction.

Sense of Professional Achievement (SPA) had a strong positive correlation with Work Engagement (WOE; r = 0.55), emphasizing its role as a key driver of engagement and a potential buffer against stress and exhaustion.

### 4.4 Differences in key variables among demographic groups of secondary school physical education teachers

#### 4.4.1 Gender differences in key variables

Gender differences in key variables were analyzed using independent samples t-tests, with the results summarized in **Table 6**. Significant differences were observed across all variables, suggesting that male and female physical education teachers experience distinct levels of competency, stress, exhaustion, achievement, and engagement.

**Table 6:** Gender differences in key variables among secondary school physical education teachers.

Variables	Gender	M	SD	t	*P*
Teacher competency	Male	3.526	0.600	6.988	0.000
	Female	3.291	0.628		
Occupational stress	Male	3.171	0.592	7.919	0.000
	Female	2.912	0.605		
Emotional exhaustion	Male	2.732	0.805	-2.507	0.012
	Female	2.841	0.786		
Sense of professional achievement	Male	3.273	0.650	3.713	0.000
	Female	3.144	0.624		
Work engagement	Male	3.648	0.618	4.819	0.000
	Female	3.485	0.623		

Male teachers reported significantly higher competency levels (M = 3.526, SD = 0.600) than female teachers (M = 3.291, SD = 0.628; t = 6.988, p < 0.001). This indicates that male teachers may perceive themselves as more skilled in integrating knowledge, skills, and behaviors required for effective teaching.

Male teachers also experienced higher occupational stress (M = 3.171, SD = 0.592) compared to their female counterparts (M = 2.912, SD = 0.605; t = 7.919, p < 0.001). This result suggests that male teachers might encounter more job-related challenges, including physical demands and administrative responsibilities.

Female teachers exhibited slightly higher emotional exhaustion (M = 2.841, SD = 0.786) than male teachers (M = 2.732, SD = 0.805; t = -2.507, p = 0.012). This finding highlights the greater psychological toll potentially faced by female teachers in managing professional demands.

Male teachers reported significantly higher levels of professional achievement (M = 3.273, SD = 0.650) compared to female teachers (M = 3.144, SD = 0.624; t = 3.713, p < 0.001). This suggests that male teachers may derive greater satisfaction from their accomplishments and self-efficacy in the profession.

Male teachers also showed higher work engagement (M = 3.648, SD = 0.618) than female teachers (M = 3.485, SD = 0.623; t = 4.819, p < 0.001). This implies that male teachers might demonstrate greater enthusiasm, energy, and dedication to their teaching roles.

#### 4.4.2 Age differences in key variables

Age differences in key variables were analyzed using one-way ANOVA, and the results are presented in **Table 7**. Significant differences were observed in some variables across age groups, while others showed no statistically significant variation.

**Table 7:** Age differences in key variables among secondary school physical education teachers.

Variables	Age	M	SD	F	*P*
Teacher competency	≤30	3.417	0.625	0.309	0.819
	31-40	3.395	0.629		
	41-50	3.420	0.628		
	>50	3.349	0.588		
Occupational stress	≤30	3.057	0.590	5.342	0.001
	31-40	3.085	0.605		
	41-50	2.996	0.628		
	>50	2.779	0.643		
Emotional exhaustion	≤30	2.751	0.787	2.265	0.079
	31-40	2.854	0.799		
	41-50	2.741	0.801		
	>50	2.693	0.784		
Sense of professional achievement	≤30	3.228	0.619	2.070	0.102
	31-40	3.158	0.649		
	41-50	3.243	0.639		
	>50	3.307	0.673		
Work engagement	≤30	3.586	0.620	0.952	0.415
	31-40	3.532	0.632		
	41-50	3.583	0.627		
	>50	3.633	0.604		

No significant age differences were found for teacher competency (F = 0.309, p = 0.819). This suggests that perceived competency levels are consistent across age groups, indicating that experience and age do not necessarily influence teachers’ self-perception of their skills and abilities.

Significant age differences were found for occupational stress (F = 5.342, p = 0.001). Teachers aged ≤30 reported the highest levels of occupational stress (M = 3.057, SD = 0.590), followed by those aged 31–40 (M = 3.085, SD = 0.605). Stress levels decreased in the older groups, with the lowest levels observed in teachers aged >50 (M = 2.779, SD = 0.643). This trend indicates that younger teachers may face more challenges in managing their professional responsibilities and workload compared to their older, more experienced colleagues.

Although differences in emotional exhaustion across age groups approached significance (F = 2.265, p = 0.079), the results were not statistically significant. However, a trend was observed where teachers aged ≤30 reported slightly higher emotional exhaustion (M = 2.751, SD = 0.787) than those in older age groups, with the lowest levels observed in teachers aged >50 (M = 2.693, SD = 0.784).

No significant differences were found for the sense of professional achievement across age groups (F = 2.070, p = 0.102). This indicates that teachers, regardless of their age, experience similar levels of fulfillment and self-efficacy in their professional roles.

No significant age differences were found for work engagement (F = 0.952, p = 0.415). Teachers across all age groups reported comparable levels of vigor, dedication, and absorption in their work.

#### 4.4.3 Education level differences in key variables

Education level differences in key variables were analyzed using one-way ANOVA, and the results are presented in **Table 8**. No statistically significant differences were observed across educational levels (junior college and below, bachelor, graduate) for the measured variables, although some trends are worth noting.

**Table 8:** Education level differences in key variables among secondary school physical education teachers.

Variables	Education level	M	SD	F	*P*
Teacher competency	Junior college and below	3.426	0.618	2.771	0.063
	Bachelor	3.369	0.624		
	Graduate	3.464	0.632		
Occupational stress	Junior college and below	3.041	0.625	1.984	0.138
	Bachelor	3.064	0.611		
	Graduate	2.982	0.601		
Emotional exhaustion	Junior college and below	2.802	0.766	1.772	0.179
	Bachelor	2.814	0.814		
	Graduate	2.717	0.787		
Sense of professional achievement	Junior college and below	3.248	0.651	2.421	0.089
	Bachelor	3.170	0.640		
	Graduate	3.245	0.627		
Work engagement	Junior college and below	3.600	0.636	1.238	0.290
	Bachelor	3.539	0.625		
	Graduate	3.585	0.618		

Although no statistically significant differences were found (F = 2.771, p = 0.063), teachers with graduate-level education reported slightly higher perceived competency (M = 3.464, SD = 0.632) compared to those with bachelor-level education (M = 3.369, SD = 0.624) and those with junior college and below (M = 3.426, SD = 0.618). This trend suggests that higher educational attainment may be associated with greater confidence in teaching competencies.

No significant differences in occupational stress were observed across education levels (F = 1.984, p = 0.138). However, teachers with junior college and below reported the highest levels of stress (M = 3.041, SD = 0.625), while those with graduate degrees reported the lowest levels (M = 2.982, SD = 0.601). This suggests a potential trend where higher education may buffer occupational stress.

No significant differences were found for emotional exhaustion across education levels (F = 1.772, p = 0.179). Interestingly, teachers with junior college and below reported slightly higher emotional exhaustion (M = 2.802, SD = 0.766) compared to those with graduate degrees (M = 2.717, SD = 0.787).

Differences in sense of professional achievement approached significance (F = 2.421, p = 0.089), with teachers holding a bachelor’s degree reporting the highest levels (M = 3.170, SD = 0.640), slightly above those with junior college and below (M = 3.248, SD = 0.651) and graduate degrees (M = 3.245, SD = 0.627).

No significant differences in work engagement were observed (F = 1.238, p = 0.290), though teachers with junior college and below reported the highest engagement levels (M = 3.600, SD = 0.636), compared to those with bachelor (M = 3.539, SD = 0.625) and graduate degrees (M = 3.585, SD = 0.618).

#### 4.4.4 Professional title differences in key variables

Professional title differences in key variables among secondary school physical education teachers were examined using one-way ANOVA, and the results are presented in **Table 9**. Significant differences were observed for several variables, highlighting the influence of professional title on teacher experiences.

**Table 9:** Professional title differences in key variables among secondary school physical education teachers.

Variables	Professional title	M	SD	F	*P*
Teacher competency	No title	3.404	0.615	1.664	0.173
	primary	3.373	0.632		
	Intermediate	3.492	0.599		
	Senior	3.427	0.797		
Occupational stress	No title	3.188	0.591	25.115	0.000
	primary	2.928	0.598		
	Intermediate	2.852	0.584		
	Senior	2.764	0.715		
Emotional exhaustion	No title	2.854	0.798	3.206	0.022
	primary	2.738	0.796		
	Intermediate	2.686	0.773		
	Senior	2.855	0.846		
Sense of professional achievement	No title	3.083	0.617	17.027	0.000
	primary	3.275	0.652		
	Intermediate	3.402	0.588		
	Senior	3.378	0.710		
Work engagement	No title	3.530	0.630	1.244	0.292
	primary	3.592	0.626		
	Intermediate	3.608	0.602		
	Senior	3.564	0.669		

No statistically significant differences in teacher competency were observed across professional titles (F = 1.664, p = 0.173). However, teachers with intermediate titles reported the highest competency scores (M = 3.492, SD = 0.599), followed by those with senior titles (M = 3.427, SD = 0.797). Teachers with no titles and primary titles reported slightly lower competency levels (M = 3.404, SD = 0.615; M = 3.373, SD = 0.632, respectively).

Significant differences in occupational stress were found among teachers with different professional titles (F = 25.115, p < 0.001). Teachers with no titles reported the highest levels of stress (M = 3.188, SD = 0.591), while those with senior titles reported the lowest stress levels (M = 2.764, SD = 0.715). Teachers with primary and intermediate titles fell in between (M = 2.928, SD = 0.598; M = 2.852, SD = 0.584, respectively). This indicates that advanced professional titles may provide a buffer against occupational stress.

Emotional exhaustion also differed significantly across professional titles (F = 3.206, p = 0.022). Teachers with no titles reported the highest levels of emotional exhaustion (M = 2.854, SD = 0.798), followed by those with senior titles (M = 2.855, SD = 0.846). Teachers with primary and intermediate titles reported slightly lower exhaustion levels (M = 2.738, SD = 0.796; M = 2.686, SD = 0.773, respectively).

Professional title significantly influenced teachers’ sense of professional achievement (F = 17.027, p < 0.001). Teachers with intermediate titles reported the highest levels of professional achievement (M = 3.402, SD = 0.588), followed by those with senior titles (M = 3.378, SD = 0.710) and primary titles (M = 3.275, SD = 0.652). Teachers with no titles reported the lowest sense of professional achievement (M = 3.083, SD = 0.617).

No significant differences in work engagement were observed across professional titles (F = 1.244, p = 0.292). However, teachers with senior titles reported the highest engagement levels (M = 3.564, SD = 0.669), while teachers with no titles reported slightly lower engagement levels (M = 3.530, SD = 0.630).

#### 4.4.5 School location differences in key variables

Differences in key variables between rural and urban secondary school physical education teachers were examined using independent sample t-tests. The results are summarized in **Table 10**.

**Table 10:** School location differences in key variables among secondary school physical education teachers.

Variables	Gender	M	SD	t	*P*
Teacher competency	Rural	3.408	0.631	0.150	0.881
	Urban	3.403	0.620		
Occupational stress	Rural	3.023	0.612	-0.917	0.359
	Urban	3.054	0.613		
Emotional exhaustion	Rural	2.736	0.798	-2.420	0.016
	Urban	2.841	0.793		
Sense of professional achievement	Rural	3.205	0.639	-0.101	0.919
	Urban	3.209	0.642		
Work engagement	Rural	3.574	0.628	0.564	0.573
	Urban	3.555	0.624		

No significant differences were observed in teacher competency between teachers from rural (M = 3.408, SD = 0.631) and urban schools (M = 3.403, SD = 0.620) (t = 0.150, p = 0.881). This suggests that location does not significantly influence teachers’ perceptions of their competency.

Similarly, no significant differences were found in occupational stress between rural (M = 3.023, SD = 0.612) and urban school teachers (M = 3.054, SD = 0.613) (t = -0.917, p = 0.359), indicating comparable stress levels across locations.

A significant difference was found in emotional exhaustion, with rural school teachers reporting lower levels (M = 2.736, SD = 0.798) than urban school teachers (M = 2.841, SD = 0.793) (t = -2.420, p = 0.016). This indicates that urban school teachers may experience slightly higher emotional exhaustion than their rural counterparts.

No significant differences were observed in the sense of professional achievement between rural (M = 3.205, SD = 0.639) and urban school teachers (M = 3.209, SD = 0.642) (t = -0.101, p = 0.919), suggesting that teachers’ sense of professional achievement is similar across school locations.

There were no significant differences in work engagement between rural (M = 3.574, SD = 0.628) and urban school teachers (M = 3.555, SD = 0.624) (t = 0.564, p = 0.573), indicating comparable levels of engagement in both groups.

### 4.5 Test results of mediation effects

The structural equation model examining the relationships among teacher competency, occupational stress, emotional exhaustion, sense of professional achievement, and work engagement demonstrated a good model fit. As shown in **Table 11**, the values for χ²/*df* (4.013), CFI (0.966), TLI (0.958), SRMR (0.041), and RMSEA (0.047, 90% CI: 0.043–0.052) indicate that the model adequately represents the observed data.

**Table 11:** Questionnaire model fitting indicators.

Model Fit	χ 2/*df*	CFI	TLI	SRMR	RMSEA (90%CI)
Model	4.013	0.966	0.958	0.041	0.047 (0.043-0.052)

**Figure 3:**
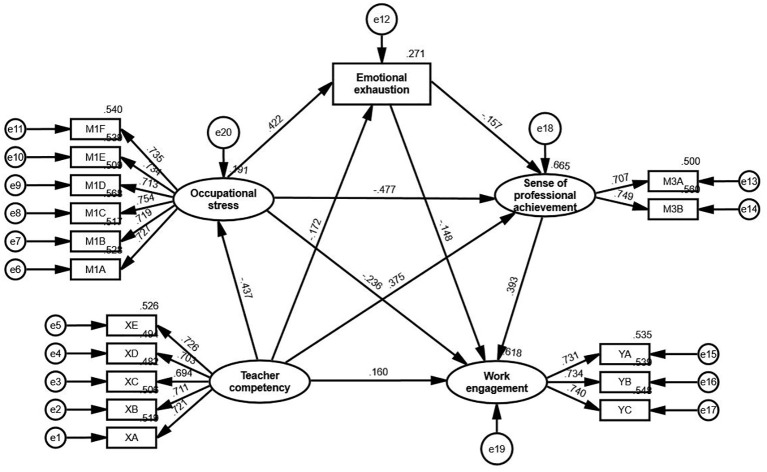
Structural equation model of the relationship between teacher competency and work engagement, with occupational stress, emotional exhaustion and sense of professional achievement as mediators. XA to XE represent the five dimensions of teacher competency (Research and Innovation Ability, Information Gathering and Learning Ability, Teaching and Organizational Ability, Professional Knowledge, Personal Characteristics). YA to YC represent the three dimensions of work engagement (Vigor, Dedication, Absorption). M1A to M1F represent the six dimensions of occupational stress (Examination Stress, Student Factors, Need for Self-Development, Family and Interpersonal Relationships, Workload, Career Expectations). M3A and M3B represent the two dimensions of sense of professional achievement (Experience of Success, Self-Efficacy). All paths are significant at the 0.01 level.

The relationships among these variables are depicted in **Figure 3**, which illustrates the pathways through which teacher competency influences work engagement, both directly and indirectly, via occupational stress, emotional exhaustion, and sense of professional achievement. All paths were statistically significant at the 0.01 level, supporting the hypothesized mediating mechanisms.

The direct, indirect, and total effects are summarized in **Table 12**. Below, the results are discussed in the context of each hypothesis:

**Table 12:** Total, direct and indirect effects in the multiple mediator model.

Path	Estimated effect	Boot SE	P	Boot LLCI	Boot ULCI	Ratio
Direct effect						
TEC→WOE	0.160	0.040	0.003	0.089	0.220	28.27%
Indirect effects						71.73%
TEC→OCS→WOE	0.103	0.021	0.005	0.075	0.149	18.20%
TEC→EME→WOE	0.025	0.007	0.007	0.016	0.038	4.42%
TEC→SPA→WOE	0.147	0.027	0.004	0.113	0.206	25.97%
TEC→OCS→EME→WOE	0.027	0.007	0.005	0.017	0.039	4.77%
TEC→OCS→SPA→WOE	0.082	0.017	0.006	0.060	0.119	14.49%
TEC→EME→SPA→WOE	0.011	0.004	0.015	0.006	0.016	1.94%
TEC→OCS→EME→SPA→WOE	0.011	0.003	0.005	0.008	0.019	1.94%
Total effect	0.566	0.024	0.000	0.528	0.607	100%

TEC, Teacher competency; OCS, Occupational stress; EME, Emotional exhaustion; SPA, Sense of professional achievement; WOE, Work engagement; Boot LLCI, the lower bound of the 95% confidence interval; Boot ULCI, the upper limit of the 95% confidence interval (Percentile Bootstrap Method with Bias Correction). The Bootstrap sample size is set at 2000.

#### 4.5.1 Direct effect (H1)

Teacher competency (TEC) had a significant direct effect on work engagement (WOE) (effect = 0.160, p = 0.003), accounting for 28.27% of the total effect. This finding supports H1, demonstrating that teacher competency directly and positively influences work engagement. The direct effect underscores the essential role of professional skills in fostering teacher engagement.

#### 4.5.2 Single-mediation effects

H2 (TEC → OCS → WOE): Occupational stress (OCS) significantly mediated the relationship between teacher competency and work engagement (effect = 0.103, p = 0.005), contributing 18.20% of the total effect. This highlights the role of stress reduction as a significant pathway through which teacher competency enhances engagement.

H3 (TEC → EME → WOE): Emotional exhaustion (EME) also mediated the relationship, though with a smaller effect (effect = 0.025, p = 0.007), accounting for 4.42% of the total effect. This emphasizes the importance of reducing emotional exhaustion to maintain engagement.

H4 (TEC → SPA → WOE): Sense of professional achievement (SPA) emerged as the strongest single mediator (effect = 0.147, p = 0.004), contributing 25.97% of the total effect. This highlights the motivational role of achievement in linking competency and engagement.

#### 4.5.3 Sequential mediation effects

H5 (TEC → OCS → EME → WOE): The sequential mediation through occupational stress and emotional exhaustion was significant (effect = 0.027, p = 0.005), accounting for 4.76% of the total effect. This pathway reflects the interconnected roles of stress and emotional resilience in shaping engagement.

H6 (TEC → OCS → SPA → WOE): Sequential mediation through occupational stress and sense of professional achievement contributed 14.49% of the total effect (effect = 0.082, p = 0.006). This pathway underscores how reducing stress enhances a sense of achievement, which in turn drives engagement.

H7 (TEC → EME → SPA → WOE): Emotional exhaustion and sense of professional achievement jointly mediated the relationship (effect = 0.011, p = 0.015), accounting for 1.94% of the total effect. This highlights the role of professional achievement in mitigating the negative impact of emotional exhaustion.

H8 (TEC → OCS → EME → SPA → WOE): The most complex sequential pathway, involving occupational stress, emotional exhaustion, and professional achievement, was significant (effect = 0.011, p = 0.005), contributing 1.94% of the total effect. This pathway demonstrates the cumulative influence of all mediators in linking teacher competency to engagement.

#### 4.5.4 Total indirect effect

The combined indirect effects of occupational stress, emotional exhaustion, and sense of professional achievement accounted for 71.73% of the total effect (effect = 0.406). This highlights the critical importance of these mediators in translating teacher competency into enhanced engagement. Among these, professional achievement played the most prominent role, but the contribution of occupational stress and emotional exhaustion further illustrates the multifaceted mechanisms underlying teacher engagement.

#### 4.5.5 Total effect

Teacher competency had a robust total effect on work engagement (effect = 0.566, p < 0.001), combining both direct and mediated pathways. These findings emphasize the importance of both personal resilience (e.g., managing stress and emotions) and external reinforcement (e.g., fostering professional achievement) in promoting teacher engagement.

### 4.6 Testing for structural invariance across gender

The structural invariance of the proposed model across gender groups was tested using multigroup structural equation modeling (MGSEM). As presented in **Table 13**, the unconstrained model exhibited acceptable fit indices (χ²/*df* = 2.506, CFI = 0.957, TLI = 0.964, SRMR = 0.037, RMSEA = 0.033), confirming a good overall model fit. Comparisons between the unconstrained model and the constrained models (measurement weights, structural weights, structural covariances, and structural residuals) revealed negligible differences in CFI and TLI (ΔCFI ≤ +0.004, ΔTLI = 0.000). These results indicate that the hypothesized structural model holds consistently across gender groups, supporting its invariance.

**Table 13:** Testing for structural invariance across gender.

	χ 2/*df*	CFI	△CFI	TLI	△TLI	SRMR	RMSEA (90%CI)
Unconstrained	2.506	0.957	–	0.964	–	0.037	0.033 (0.030-0.037)
Measurement weights	2.415	0.960	+0.003	0.964	0.000	0.038	0.032 (0.029-0.036)
Structural weights	2.408	0.960	+0.003	0.964	0.000	0.038	0.032 (0.029-0.036)
Structural covariances	2.402	0.960	+0.003	0.964	0.000	0.038	0.032 (0.029-0.036)
Structural residuals	2.375	0.961	+0.004	0.964	0.000	0.038	0.032 (0.029-0.035)

**Figure 4:**
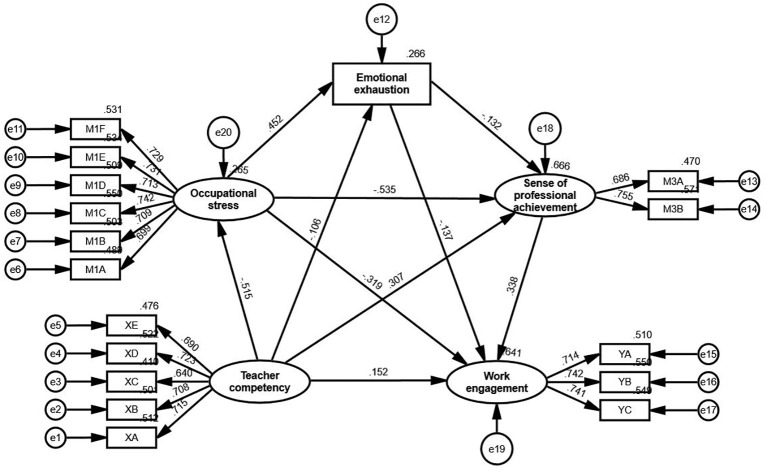
Structural equation model for male secondary school physical education teachers. All paths are significant at the 0.01 level.

**Figure 5:**
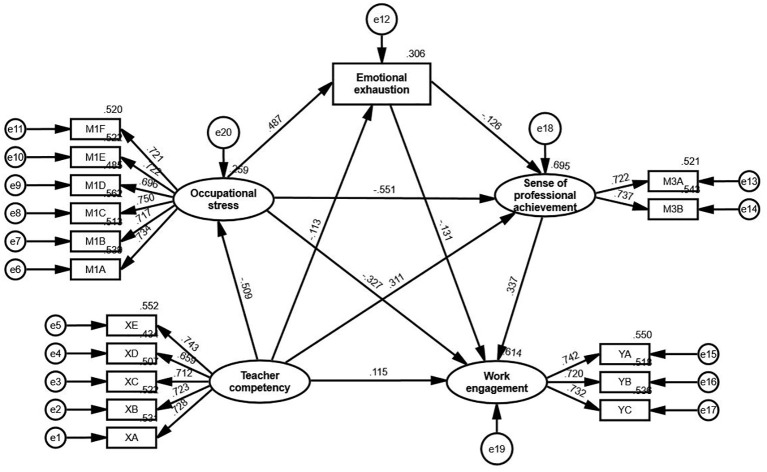
Structural equation model for female secondary school physical education teachers. All paths are significant at the 0.01 level.

**Figures 4** and **5** illustrate the structural equation models for male and female secondary school physical education teachers, respectively. All path relationships in both models were statistically significant at the 0.01 level, demonstrating that the key constructs—teacher competency, occupational stress, emotional exhaustion, sense of professional achievement, and work engagement—interact in a similar manner for both male and female teachers.

Although minor numerical variations were observed in the standardized path coefficients between genders, these differences did not impact the overall structural consistency of the model. Both male and female teachers exhibit the same underlying mechanisms through which teacher competency influences work engagement, mediated by occupational stress, emotional exhaustion, and sense of professional achievement. This consistency underscores the universal relevance of the proposed model across gender groups.

The findings validate the robustness of the hypothesized framework, highlighting its applicability across diverse populations. By demonstrating structural invariance, the study affirms that the proposed mechanisms linking teacher competency to work engagement operate consistently for male and female teachers, reinforcing the model’s generalizability.

## 5 Discussion

### 5.1 Implications of demographic differences

The results revealed significant gender differences across all key variables. Male teachers reported higher levels of teacher competency, occupational stress, sense of professional achievement, and work engagement compared to their female counterparts, while female teachers exhibited slightly higher emotional exhaustion. These findings suggest that male teachers may perceive themselves as more skilled and derive greater satisfaction from their professional accomplishments, potentially due to differing societal expectations, professional opportunities, or personal career trajectories. On the other hand, higher emotional exhaustion among female teachers highlights the psychological toll they may face, possibly stemming from additional responsibilities, such as balancing work and family roles (95, 96). These findings underscore the need for gender-sensitive interventions to reduce emotional exhaustion among female teachers while fostering greater engagement and satisfaction across both genders.

The study found significant age-related differences in occupational stress, with younger teachers experiencing higher stress levels compared to their older colleagues. This trend is likely attributable to the challenges younger teachers face in adapting to their roles, managing heavy workloads, and developing effective coping mechanisms. Conversely, older teachers may benefit from greater experience and professional stability, enabling them to manage stress more effectively (97, 98). Although emotional exhaustion and work engagement did not exhibit significant age differences, the observed trends suggest that younger teachers may require additional support during the early stages of their careers. Mentorship programs, stress management training, and tailored professional development opportunities could help alleviate these challenges and enhance well-being among younger educators.

Although no statistically significant differences were observed across educational levels for most variables, trends indicate that teachers with higher educational qualifications tend to report slightly higher perceived competency and lower levels of occupational stress. These trends suggest that advanced education may equip teachers with better skills, knowledge, and coping strategies to navigate the challenges of their profession (99). Educational institutions could leverage this insight by providing targeted training and professional development opportunities for teachers with lower educational qualifications, thereby enhancing their competencies and reducing stress.

Professional titles demonstrated significant influences on occupational stress, emotional exhaustion, and sense of professional achievement. Teachers with senior or intermediate titles reported lower stress levels, less emotional exhaustion, and higher professional achievement compared to their counterparts with no titles or primary titles. These differences likely reflect the benefits of greater recognition, experience, and stability associated with advanced titles. These findings emphasize the importance of creating pathways for career advancement and recognizing teachers’ contributions, which could serve as motivational drivers for reducing stress and enhancing engagement.

The findings showed no significant differences in most variables between rural and urban teachers, except for emotional exhaustion, where urban teachers reported slightly higher levels. This difference may reflect the unique challenges faced by urban teachers, such as larger class sizes, greater administrative demands, or higher expectations for student outcomes (100, 101). These findings highlight the importance of considering contextual factors when designing interventions to support teachers’ well-being and engagement. Urban schools, in particular, may benefit from additional resources and support systems to help teachers manage their workload and reduce emotional exhaustion.

### 5.2 Teacher competency and work engagement

The findings confirm H1, demonstrating that teacher competency exerts a significant direct effect on work engagement. This result highlights that teachers with higher competency—characterized by research and organizational abilities, professional knowledge, and personal traits—are better equipped to sustain enthusiasm, dedication, and absorption in their teaching roles. These findings align with prior literature emphasizing the critical role of teacher competency in enhancing classroom performance (102–104); however, this study extends existing research by quantifying its direct impact on engagement within the unique context of physical education (PE) teachers. This distinction is particularly important because it addresses how teacher competency shapes not only performance outcomes but also the psychological states that underpin effective teaching, such as intrinsic motivation and sustained focus.

The implications of these findings are substantial. They underscore the importance of targeted professional development initiatives aimed at enhancing teacher competency as a mechanism for fostering work engagement. Unlike generic professional development programs, these efforts should prioritize practical, context-specific competencies that address the unique challenges faced by PE teachers. For example, while prior research often highlights general teaching skills (105), our findings suggest that competency development should also encompass specialized training in effective activity design, physical safety protocols, and techniques for fostering student engagement tailored to the physical education environment. Such targeted initiatives can help PE teachers feel more confident and capable in their roles, which, in turn, promotes greater engagement and enthusiasm for their work.

Furthermore, the findings support the notion that teacher competency contributes to work engagement by fostering a sense of mastery and self-efficacy, which are key drivers of intrinsic motivation. Teachers who perceive themselves as competent are more likely to approach their roles with energy and commitment, creating a virtuous cycle of positive professional attitudes and behaviors (106, 107). This is particularly relevant for PE teachers, whose work often involves unique physical and logistical demands that require both technical expertise and strong organizational skills. By equipping teachers with these competencies, schools and policymakers can directly enhance not only pedagogical outcomes but also the overall well-being and professional satisfaction of teachers.

Finally, these findings emphasize the broader implications of teacher competency for educational systems. Beyond the individual benefits for teachers, fostering work engagement through competency development can have positive ripple effects on student outcomes, classroom dynamics, and overall school performance. For instance, engaged teachers are more likely to create dynamic, interactive learning environments that inspire students and promote active participation (108). This underscores the need for educational institutions to view competency development as a strategic investment that supports both teacher and student success.

### 5.3 Mediation effects of occupational stress, emotional exhaustion, and sense of professional achievement

The mediation analysis provides in-depth insights into how teacher competency influences work engagement indirectly, as hypothesized in H2, H3, and H4. Each mediator—occupational stress, emotional exhaustion, and sense of professional achievement—captures a unique dimension of the teaching experience, offering a comprehensive understanding of the mechanisms underlying teacher engagement.

Occupational stress was found to be a significant mediator, accounting for 18.20% of the total effect, as posited in H2. Teachers with higher competency levels appear better equipped to manage stressors such as examination pressures, interpersonal challenges, and career-related demands. This finding aligns with the Job Demands-Resources (JD-R) model, which emphasizes the buffering role of personal resources, such as competency, in mitigating the negative impact of job demands (47). Reduced occupational stress not only alleviates teachers’ psychological burden but also enhances their ability to focus and engage in their professional roles (109). These results highlight the interplay between teacher competency and stress, underscoring the importance of fostering professional environments that support stress management and resource optimization.

Emotional exhaustion was identified as a second mediator, accounting for 4.42% of the total effect, as hypothesized in H3. While its mediating role is less pronounced compared to occupational stress, it highlights the cumulative psychological toll of teaching-related demands. Teachers with lower competency levels are more susceptible to emotional exhaustion, which, in turn, impairs their ability to sustain work engagement (29). This finding underscores the importance of addressing emotional exhaustion as a key barrier to teacher engagement. Emotional resilience, fostered through targeted support and well-being initiatives, could play a pivotal role in enabling teachers to cope with the demanding nature of their profession. Additionally, addressing the root causes of emotional exhaustion, such as heavy workloads and insufficient resources, is critical for maintaining teacher engagement.

Sense of professional achievement emerged as the strongest mediator, accounting for 25.97% of the total effect, as predicted in H4. This underscores the motivational significance of feeling accomplished in one’s work, serving as a critical link between competency and engagement (68). Teachers who perceive themselves as successful in their roles are more likely to maintain high levels of enthusiasm, commitment, and sustained engagement. The results suggest that fostering professional achievement is central to enhancing work engagement. Teachers who experience recognition for their contributions and observe tangible outcomes of their efforts are more likely to feel motivated and valued in their profession. Recognition systems, constructive feedback, and opportunities for career advancement could further enhance teachers’ sense of achievement and reinforce their engagement.

These findings collectively demonstrate the multifaceted ways in which occupational stress, emotional exhaustion, and sense of professional achievement mediate the relationship between teacher competency and work engagement. While occupational stress and emotional exhaustion highlight the negative impacts of job demands and psychological strain, sense of professional achievement emphasizes the positive motivational pathways that drive engagement. Together, these mediators provide a balanced perspective on both the challenges and opportunities within the teaching profession, underscoring the complex interplay between competency and engagement.

### 5.4 Chain mediation effects

The study further examines the complex pathways involving sequential mediators, providing deeper insights into how teacher competency influences work engagement through interconnected psychological mechanisms. These sequential mediation effects, though varying in size, highlight the dynamic interplay between occupational stress, emotional exhaustion, and sense of professional achievement in shaping work engagement.

H5: Sequential Mediation of Occupational Stress and Emotional Exhaustion

The sequential mediation pathway involving occupational stress and emotional exhaustion accounts for 4.77% of the total effect. This pathway demonstrates that reducing occupational stress indirectly prevents emotional exhaustion, thereby enhancing engagement. While modest in size, this finding underscores the cascading impact of stress on emotional well-being and engagement. It highlights the interdependence of psychological factors in work settings, where alleviating stress not only reduces its immediate impact but also mitigates downstream emotional exhaustion. This result aligns with the Job Demands-Resources (JD-R) model (47), reinforcing the importance of addressing stress as a fundamental step in improving teacher engagement.

H6: Sequential Mediation of Occupational Stress and Sense of Professional Achievement

The pathway involving occupational stress and sense of professional achievement contributes a more substantial portion of the total effect (14.49%). This finding underscores the dual benefits of stress management: enhancing psychological well-being and fostering professional satisfaction. Teachers who effectively manage stress are more likely to experience a sense of accomplishment and fulfillment in their roles, which in turn amplifies their engagement (110). This pathway emphasizes the motivational significance of professional achievement as an outcome of reduced stress (111, 112), suggesting that interventions targeting stress reduction can have far-reaching effects on engagement by simultaneously boosting teachers’ sense of professional success.

H7: Sequential Mediation of Emotional Exhaustion and Sense of Professional Achievement

The sequential mediation pathway involving emotional exhaustion and sense of professional achievement accounts for 1.94% of the total effect. This pathway illustrates how emotional exhaustion, though detrimental, can influence engagement indirectly by impacting teachers’ sense of achievement. Teachers experiencing lower emotional exhaustion are more likely to feel successful in their roles, which in turn fosters greater engagement. While smaller in effect size, this pathway highlights the intricate connection between psychological states and motivational outcomes, emphasizing the importance of addressing both emotional resilience and professional satisfaction in teacher support initiatives.

H8: Full Chain Mediation of Occupational Stress, Emotional Exhaustion, and Sense of Professional Achievement

The most complex pathway, involving the combined mediation of occupational stress, emotional exhaustion, and sense of professional achievement, accounts for 1.94% of the total effect. Although this effect size is relatively small, it provides a comprehensive perspective on how multiple mediators interact sequentially to influence engagement. This pathway highlights the cumulative impact of reducing stress, fostering emotional well-being, and enhancing professional satisfaction. While less prominent in its statistical contribution, the full mediation chain reinforces the importance of addressing these interconnected factors holistically to create a supportive and engaging work environment for teachers.

These findings reveal that the sequential mediation pathways operate through distinct yet interconnected mechanisms, reflecting the layered nature of teacher engagement. Occupational stress emerges as a critical starting point, influencing both emotional exhaustion and professional achievement. Emotional exhaustion serves as a pivotal psychological barrier, while professional achievement acts as a key motivational driver that bridges competency and engagement. Collectively, these pathways underscore the need for multi-dimensional strategies that address both the challenges (e.g., stress and exhaustion) and the drivers (e.g., professional satisfaction) of teacher engagement. By acknowledging the complexity of these mediation chains, future research and interventions can better support teachers’ well-being and professional fulfillment.

### 5.5 Structural invariance across gender

The structural invariance analysis confirmed that the hypothesized model demonstrates consistent relationships across male and female teachers. This finding underscores the robustness and universality of the structural model in explaining how teacher competency, occupational stress, emotional exhaustion, sense of professional achievement, and work engagement interact, regardless of gender.

The consistent pathways observed across genders highlight that the mechanisms linking teacher competency to work engagement through occupational stress, emotional exhaustion, and sense of professional achievement operate similarly for both male and female teachers. This structural alignment suggests that the foundational constructs in the model—such as stress management, emotional well-being, and professional satisfaction—are equally relevant for understanding work engagement across genders. The invariance further supports the generalizability of the theoretical framework, reinforcing its applicability in diverse teaching contexts.

Although minor numerical differences were observed in pathway strengths, these did not alter the overall structural consistency between genders. Both male and female teachers benefit from reduced occupational stress, which mitigates emotional exhaustion and enhances professional achievement, ultimately driving higher work engagement. Similarly, the motivational role of professional achievement and the buffering effects of personal resources like teacher competency remain critical factors across genders, reflecting the shared psychological processes underlying engagement.

By affirming structural consistency, these findings validate the universal relevance of teacher competency and its mediators in fostering work engagement. This consistency supports the application of the model to develop strategies aimed at improving teacher well-being and engagement, regardless of gender. Future research could explore additional contextual or cultural factors that may interact with these mechanisms to further refine the understanding of teacher engagement.

### 5.6 Implications for educational management and policy

#### 5.6.1 Teacher well-being and professional development

The findings emphasize the critical importance of teacher competency in fostering well-being and engagement. Teachers with higher competency reported reduced occupational stress, greater professional achievement, and higher levels of engagement. Professional development programs should therefore focus on enhancing teacher competency not only through technical skill-building but also by addressing psychological and emotional resilience (1, 8, 113). For physical education (PE) teachers, who face unique physical and psychological demands, targeted initiatives that integrate stress management, mental health support, and recognition of professional contributions are essential for sustaining career satisfaction and engagement over time.

#### 5.6.2 Implications for student outcomes and public health

Engaged teachers play a pivotal role in shaping student outcomes, particularly in physical education, where they serve as role models for promoting teamwork, discipline, and healthy lifestyle choices (1, 42). This study highlights the role of occupational stress and emotional exhaustion as critical barriers to engagement, which can undermine teachers’ ability to positively influence students. Addressing these barriers is not only crucial for improving teaching effectiveness but also for advancing public health goals, such as reducing sedentary behaviors and obesity among adolescents (114). By supporting teacher engagement, educational policies can achieve broader societal benefits that extend well beyond the classroom.

#### 5.6.3 Organizational support and resource allocation

Educational institutions must adopt systemic approaches to alleviate occupational stress and support teacher well-being. Measures such as reducing excessive workloads, clarifying role expectations, and fostering supportive school cultures can help mitigate stress and emotional exhaustion (7, 8). Structured peer support programs and recognition systems that celebrate teachers’ achievements are vital for sustaining engagement (45, 46). Moreover, adequate funding and resources for PE programs—often undervalued in the broader educational system—are essential for enabling teachers to effectively perform their roles and maintain professional satisfaction.

#### 5.6.4 Addressing demographic variations

The observed demographic differences in key variables provide critical insights for tailoring interventions. Gender differences, for instance, revealed that male teachers reported higher competency, occupational stress, professional achievement, and engagement, while female teachers experienced slightly higher emotional exhaustion. These findings suggest the need for gender-sensitive approaches. Female teachers may benefit from initiatives that prioritize stress management and emotional resilience, such as flexible work arrangements and mental health support (12, 80). Male teachers, on the other hand, may be more motivated by recognition of their professional accomplishments and opportunities for leadership development (17, 45).

Age differences also highlighted that younger teachers experienced higher occupational stress compared to their older colleagues, suggesting the importance of mentorship programs and early-career support to help new teachers manage professional challenges. Additionally, teachers with senior professional titles reported lower stress levels and higher professional achievement compared to those with no titles or primary titles. This underscores the motivational role of professional recognition and the need to create clear pathways for career advancement.

Finally, differences between rural and urban teachers suggest that context-specific policies are necessary (45, 60). Urban teachers, who reported higher levels of emotional exhaustion, may benefit from targeted support addressing the unique demands of urban teaching environments, such as large class sizes or administrative workloads (9, 59).

#### 5.6.5 Contributions to educational policy

The interconnected nature of the factors influencing teacher engagement underscores the need for comprehensive and integrated policy frameworks. Policymakers should focus on aligning teacher competency development with broader stress-reduction strategies and professional recognition mechanisms. Resources should be allocated to continuous professional training, institutional reforms to reduce unnecessary job demands, and the establishment of teacher well-being programs. These policies will strengthen teacher support systems, enhance educational outcomes, and contribute to a sustainable teaching workforce.

## 6 Limitations and future directions

This study has several limitations that merit careful consideration and provide opportunities for future research. First, the reliance on self-reported data introduces potential biases, such as social desirability and common method variance. Although statistical tests were conducted to address these issues, future research could enhance the validity of findings by incorporating objective measures, such as observational data, administrative records, or physiological indicators of stress.

Second, the cross-sectional design of the study limits the ability to infer causality among teacher competency, work engagement, and the mediating variables. Longitudinal studies are strongly recommended to better understand the temporal dynamics and potential bidirectional relationships among these factors, providing deeper insights into their interplay over time.

Third, the study focuses exclusively on secondary school physical education teachers within a specific geographic region, which may restrict the generalizability of the findings to other contexts. To address this, future research should seek to replicate and extend these results in diverse educational settings, cultural contexts, and across teachers from different subject areas and grade levels to ensure broader applicability and validity.

Additionally, exploring other potential mediating and moderating variables could deepen the understanding of teacher well-being and engagement. For example, cultural norms, school leadership styles, organizational support, and intrinsic motivations may play critical roles in shaping teachers’ professional experiences. Qualitative approaches, such as in-depth interviews or focus groups, could complement quantitative findings by capturing richer, context-specific insights into teachers’ lived experiences.

Lastly, future research should focus on translating these findings into actionable interventions. Specifically, studies could evaluate the effectiveness of stress management training, resilience-building programs, and professional recognition initiatives in addressing the mediators identified in this study. These strategies could provide practical solutions to enhance teacher well-being, foster engagement, and ultimately improve student outcomes.

## 7 Conclusion

This study highlights important demographic differences in key variables among secondary school physical education teachers, such as higher competency, achievement, and engagement reported by male teachers, while female teachers experienced greater emotional exhaustion. Younger teachers faced higher occupational stress, and professional titles influenced stress levels and achievement. Additionally, the analysis confirms the pivotal role of teacher competency in fostering work engagement, both directly and through the mediating effects of occupational stress, emotional exhaustion, and professional achievement. Notably, structural invariance across gender groups validates the universal applicability of the proposed model, emphasizing its robustness and relevance for diverse teacher populations.

## Data Availability

The original contributions presented in the study are included in the article/**Supplementary Material**. Further inquiries can be directed to the corresponding author.
